# Studying the potential impact of automated document classification on scheduling a systematic review update

**DOI:** 10.1186/1472-6947-12-33

**Published:** 2012-04-19

**Authors:** Aaron M Cohen, Kyle Ambert, Marian McDonagh

**Affiliations:** 1 Department of Medical Informatics and Clinical Epidemiology, Oregon Health & Science University, Portland, OR, USA

## Abstract

**Background:**

Systematic Reviews (SRs) are an essential part of evidence-based medicine, providing support for clinical practice and policy on a wide range of medical topics. However, producing SRs is resource-intensive, and progress in the research they review leads to SRs becoming outdated, requiring updates. Although the question of how and when to update SRs has been studied, the best method for determining when to update is still unclear, necessitating further research.

**Methods:**

In this work we study the potential impact of a machine learning-based automated system for providing alerts when new publications become available within an SR topic. Some of these new publications are especially important, as they report findings that are more likely to initiate a review update. To this end, we have designed a classification algorithm to identify articles that are likely to be included in an SR update, along with an annotation scheme designed to identify the most important publications in a topic area. Using an SR database containing over 70,000 articles, we annotated articles from 9 topics that had received an update during the study period. The algorithm was then evaluated in terms of the overall correct and incorrect alert rate for publications meeting the topic inclusion criteria, as well as in terms of its ability to identify important, update-motivating publications in a topic area.

**Results:**

Our initial approach, based on our previous work in topic-specific SR publication classification, identifies over 70% of the most important new publications, while maintaining a low overall alert rate.

**Conclusions:**

We performed an initial analysis of the opportunities and challenges in aiding the SR update planning process with an informatics-based machine learning approach. Alerts could be a useful tool in the planning, scheduling, and allocation of resources for SR updates, providing an improvement in timeliness and coverage for the large number of medical topics needing SRs. While the performance of this initial method is not perfect, it could be a useful supplement to current approaches to scheduling an SR update. Approaches specifically targeting the types of important publications identified by this work are likely to improve results.

## Background

Evidence-based medicine (EBM) is the process of applying the best available evidence gained from clinical research to the practice of medicine [1]. While this is certainly a desirable goal, a typical physician’s heavy workload can make it difficult to realize. Practicing physicians may not have time to consult the primary literature to identify the best-available evidence for each and every patient. Therefore the actual practice of EBM is dependent upon clinicians having access to syntheses of the best-available primary evidence applicable to their patients. These syntheses, such as systematic reviews (SRs), make the available evidence more accessible and usable in clinical practice. The Cochrane Collaboration states that an SR:

*"“…attempts to collate all empirical evidence that fits pre-specified eligibility criteria in order to answer a specific research question. It uses explicit, systematic methods that are selected with a view to minimizing bias, thus providing more reliable findings from which conclusions can be drawn and decisions made*[2].”

SRs are literature reviews designed to locate, appraise and synthesize the best-available evidence from clinical studies of diagnosis, treatment, prognosis, or etiology, and provide informative empirical answers to specific medical questions. SRs inform medical recommendations, guiding both practice and policy, such as in the creation of published practice guidelines [3].

The process of creating and maintaining SRs is resource- and labor-intensive, typically requiring 6–12 months of effort, with the main expense being personnel time. There is ample evidence that SRs become outdated as research progresses, and thus need to be periodically updated [4,5]. Best practice in medicine is continually changing, requiring incorporation of new information as it becomes available, so SRs must undergo periodic updates in order to remain useful and accurate. Updates are costly in terms of both time and money, and can take as much time and effort as the original SR [6]. Typically SR programs, such as the Drug Effectiveness Review Project (DERP) can only assess a past SR topic for new literature once or twice a year, leading to a 6–12 month lag in recognizing new evidence and beginning the planning of an SR update.

Although there exists research guidance on when and how to update SRs [6,7], the process is not well understood. A comparison by Shekelle of two methods (known as RAND and Ottawa) for determining the need for an SR to be updated found that both begin with an initial literature search [8]. Neither method provides guidance on *when* to conduct the required literature search. The machine learning method proposed here provides exactly this guidance, and fits into the SR update process ahead of review commitment decision methods such as those assessed by Shekelle.

A survival analysis study of SRs by Shojania [5] found that the median duration of an SR not needing an update was 5.5 years. However, there was quite a lot of variation around this median – 23% of reviews needed an update within 2 years, and 15% within just 1 year of publication. While a more active SR research topic area would logically require more frequent updates, Shojania also found that areas with more heterogenous research tended to require more frequent updates as well, because new evidence is more likely to alter the previous findings by reducing the variation across results. Clearly, there is a strong need for informatics support in determining when an SR topic is due for an update.

Building on our prior work in applying automated document classification to work prioritization for SRs [9-11], in this paper we perform an initial investigation of the potential impact of automated document classification to the SR logistical process. While other researchers have investigated the use of machine learning in supporting EBM, most notably Aphinyanaphongs [12], Kilicoglu [13,14], and Matwin [15], this is the first study that we are aware of which specifically looks at the impact of machine learning methods on SR update scheduling. We seek to study the potential effect of automated document classification on the process of SR update, in terms of need recognition, planning, and scheduling.

Here, we define a document classification task called *New Update Alert*. The idea behind *New Update Alert* is that as publications become available to the SR team, an automated document classification system may be able to determine which ones are most likely to be included in the SR update. When an article is detected that is likely to be included in the SR update, the system alerts the SR leader, perhaps via an automatically generated email message, or using a custom RSS (Really Simple Syndication) feed. For the purposes of this work, a publication becomes available to the review team when it is indexed in MEDLINE, and therefore is findable using the search queries previously designed for the SR topic. The algorithm looks at each article meeting the original review search criteria, and notifies the team about articles that it predicts as likely to be included in an update.

We define a correct alert to be one that notifies the SR team about a publication that will be included in the eventual SR report update. These are publications that include new evidence regarding interventions, populations, or study designs relevant to the report. An incorrect alert is an alert about publication that is not eventually included in the SR update; these are “false alarms”. The machine predictions are not perfect, and a range of settings trading off sensitivity and specificity are possible. Greater sensitivity means that the team will be notified about the publication of a greater fraction of articles that will be ultimately included in the review update (true positives, *TP*), at the cost of more false alarms (false positives, *FP*). Furthermore, some publications may be more important than others, in that, in addition to being included in the final SR, they may include specific novel, or higher quality evidence that could motivate the scheduling, priority, or initiation of a review update. We specifically annotate and study these important publications in the work described below. For the work described here, alerts are trigged when any potentially included publication is detected, whether this is a motivating publication or not.

*New Update Alerts* could be useful to the process of SR in several ways. For example, the alerts could be used by the SR team to determine whether an SR needs an update, the urgency of the update, or when an update should be scheduled. Seeing potentially includable articles accumulate as they are published may be helpful in scheduling a review update. With a system providing *New Update Alerts*, reviewers could be made aware of studies potentially impacting the SR scope, conclusions, or recommendations at an earlier time. By examining the articles that result in alerts the reviewers could get a better initial idea of the quantity and quality of new information pertinent to an SR *before actually scheduling or conducting the review update*.

This would provide support for determining when to schedule an SR update. For example, whether a review update is needed as soon as possible, or could be postponed for a time. Given that the resources to conduct SRs are specialized and limited, the ability to coordinate review update scheduling across the full set of a team’s review topics would be a great advantage in best applying those resources and supporting the current needs of the practice of EBM. Furthermore, this could play an important role in obtaining funding to support the review. Since many SRs are dependent upon outside funding, new update alerts could provide the SR team lead with timely and important information to share with a funding organization.

Here we study the performance of an initial classification system for *New Update Alert*, leaving the issues surrounding exactly what kind of user interface to use with the alerts for future work.

## Methods

### Data sets

We created two separate data sets based on SR inclusion data collected by our automated SYstematic Review Information Automated Collection (SYRIAC) system, which has been described elsewhere [16]. The collection contains the titles, abstracts, and MeSH terms for over 70,000 documents that have been judged by experts for inclusion eligibility in various SRs performed for the DERP by researchers at Oregon Health & Science University’s Evidence-based Practice Center (EPC). Each review comprises hundreds to thousands of journal article judgments on a specific review topic. These topics are usually focused on drug therapy, and often are constrained to a particular class of drugs across multiple indications. In order to perform this work, we created time-segregated training and testing data sets for several SR topics. The training and testing data sets for each topic were mutually exclusive, and separated in time.

We define two specific time events in the SR process. The *End of the Report Cycle* occurs for a topic when an SR has had its peer review completed and it is published on the Internet (http://www.ohsu.edu/drugeffectiveness). The *Report Search Begins* event occurs when the first literature search for a subsequent review update is begun. We use the term *Pre-Update Period* to describe the time period between the *End of the Report Cycle* for the prior report, and the *Report Search Begins*. During this time, relevant studies and articles are published and new medical evidence accumulates. Some of these publications will eventually be included in the next report update for the topic. Relatively few expert resources are available to follow the SR topic during this period; the DERP conducts a yearly literature scan for each topic.

**Figure 1:**
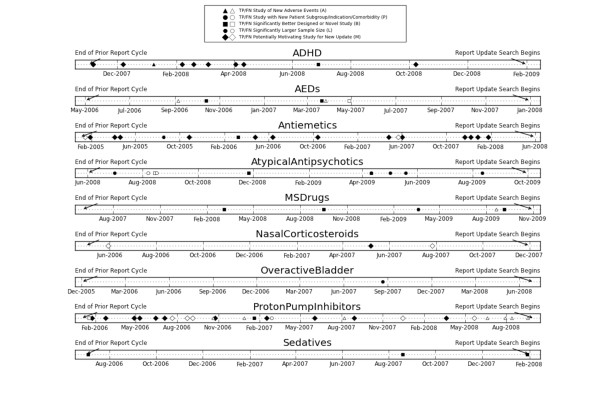
**Timeline plot of the most important studies in the inter-update period for each of the nine topics.** Black markers are publications that were correctly identified by the classification system, white markers are those that were missed. The shape of the marker designates the type of the important study as defined in the methods section.

For each topic, we used the DERP review inclusion judgments for articles with MEDLINE entry dates prior to the *End of the Report Cycle* (for the prior report) as the classification system training data for that topic. We used the DERP review inclusion judgments for articles indexed in MEDLINE during the *Pre-Update Period* (after the *End of the Report Cycle* date and prior to the *Report Search Begins* for the next update) as the testing data for that topic. Note that, for this data set, these articles were retrieved and inclusion judgments assigned after the *Pre-Update Period*, but they apply to articles that were published, indexed in MEDLINE, and are therefore potentially available to the SR team during the pre-update period. ‘Potentially available’ means that if the SR team re-ran their MEDLINE query during this time period, they would retrieve these documents, along with many others. These events and time periods are listed in temporal order and defined in Table 1 (see Figure 1 for the actual corresponding dates for each of the studied topics).

**Table 1:** Definition of temporal events and periods of a systematic review update relevant to this study

**Name**	**Type**	**Definition**
End of the Report Cycle	Event	A report has had its peer review completed and is published on the Internet.
Pre-update Period	Time Period	Between the End of the Report Cycle and the Report Search for a report update. Little work on the topic, beyond a yearly literature search, is conducted.
Report Update Search Begins	Event	Date on which literature search for a report update begins.
Report Update Period	Time Period	Between the Report Search Begins and the End of the Report Cycle for a report update. Most of the work of a report update is conducted during this period.

In this way, we can simulate both the data available for training the machine learning system for predicting *New Update Alerts*, as well as apply the trained models to documents made available within MEDLINE during the pre-update period. Since this work is actually being done after the report update of interest has been completed, we know which documents indexed in MEDLINE during the *Pre-Update Period* were actually included in the subsequent report update. This allows us to measure the performance of the classifier system on articles published during this period.

Of course, the above-described evaluation approach requires that we have SR topics for which both a prior report and a report update have been completed by the DERP investigators, and that we have the inclusion/exclusion judgments for these topics within these periods in our data collection window. For this work we used a cross-sectional snapshot of the SYRIAC database, incorporating all inclusion decisions made up to February 12, 2010. In reviewing the DERP records, we found that 11 topics met these requirements. For two of the topics, *Antiplatelets* and *NSAIDs* an insufficient number of newly included and/or excluded articles were found in the update (fewer than 10), preventing adequate analysis. Therefore we excluded these two topics from the present study, leaving us with nine topics. See Table 2 for a summary of the training and test data sets.

**Table 2:** Data sets used in this study

**TOPIC**	**TRAINING SET**			**TESTING SET**			
	**INCLUDED**	**EXCLUDED**	**TOTAL**	**INCLUDED**	**EXCLUDED**	**TOTAL**	**STUDIED**
*ADHD*	285	2542	2827	43	245	288	Yes
*AEDs*	96	1436	1532	39	476	515	Yes
*Antiemetics*	133	2364	2497	29	334	363	Yes
*Antiplatelets*	20	0	20	5	0	5	No
*AtypicalAntipsychotics*	659	3829	4488	67	785	852	Yes
*MSDrugs*	138	1774	1912	38	601	639	Yes
*NasalCorticosteroids*	13	82	95	15	62	77	Yes
*NSAIDs*	79	106	185	1	3	4	No
*OveractiveBladder*	103	823	926	25	229	254	Yes
*ProtonPumpInhibitors*	204	2333	2537	50	375	425	Yes
*Sedatives*	145	1657	1802	26	206	232	Yes

Eleven systematic review topics had both a prior report and an update completed within our data collection window. Included articles are those included in the final systematic review report, while excluded articles are those not included in the report. Drug Effectiveness Review Project (DERP) review inclusion judgments for articles with MEDLINE entry dates prior to the *End of the Report Cycle* (for the prior report) were used as training data for that topic. DERP review inclusion judgments for articles indexed in MEDLINE during the *Pre-Update Period* (after the *End of the Report Cycle* date and prior to the *Report Search Begins* for the next update) as the testing data for that topic.

For these nine topics, we separated out the data into training and test sets, as noted above, and annotated the test collection. We wanted to understand both the overall performance of the machine learning system on identifying publications for *New Update Alert,* as well as how the classifier performs on the “important” publications. These important publications are the ones that are most likely to motivate SR experts to decide that a new update is needed for the topic. These publications could change or influence the conclusions or recommendations of the SR. This could be due, for example, to a new study providing additional evidence for meta-analysis, or studying a new harm, or a new indication or patient population for a drug. We term these publications *update-motivating publications*, realizing that it may be an individual or a collection of these publications that provide the actual motivation to the SR expert to recommend an update on a review topic.

Therefore, we designed an annotation scheme to identify the important studies in the test collection. The scheme shown in Table 3 was designed using an iterative consensus process between the two senior authors (AC and MM), one an expert on EBM and conducting SRs, the other a researcher experienced in data set creation and annotation for biomedical machine learning. The annotation scheme includes four specific (A, P, B, and L) annotation codes, and one general (M) code intended to cover the different kinds of new evidence that an article might provide. This evidence could motivate the SR expert to consider (e.g., schedule, or try to pursue funding to support) an update of the SR topic. The annotation codes A, P, B, and L represent specifically-identified ways in which a study may contribute significant new information to the evidence base of an SR topic, and thereby potentially change the state of EBM on the topic. The M annotation represents new information that is not as uniquely impactful on its own, but combined with other information (for example, from additional articles such as other articles that meet the M annotation criteria) may also change the state of evidence on a topic and therefore increase the need for an SR update on this topic. These categories were determined in an iterative manner after discussing the types of new evidence that can contribute to a review update and examining publications from the update period of each topic. Certain aspects of the annotation definitions rely on the SR experience and expertise of the annotator. For example, “significantly larger sample size” must be interpreted by the annotator in the context of all prior studies performed in the given domain.

**Table 3:** Annotation guide for articles that were deemed to potentially motivate a review update on their topic

**Annotation**	**Description**
A	Study includes evidence on new or serious adverse events relevant to this topic.
P	Study includes new patient subgroup, new indication, or evidence specific to new comorbidity.
B	Study is notably better designed, or uses novel methods, compared to prior studies.
L	Study uses a significantly larger sample size than prior studies for this topic.
M	Study includes other significant evidence that may motivate a review update, when taken in combination with other studies.

Each article included in the actual systematic review update was analyzed and assigned either the single most descriptive annotation, or no annotation, if the article was not deemed to be potentially motivating for a review update.

We then annotated each of the publications from the *Pre-Update Period* that were included in the report update according to these criteria. The two senior authors (AMC and MM) discussed and modified the article annotation assignments until consensus was reached. The annotations were assigned before the machine learning models were created from the training data or applied to the test data. Therefore, none of the authors had prior information about the machine learning performance on the test data that could have biased annotation assignments. Only publications meeting the specific criteria given in Table 3 were annotated, while the remaining publications had no annotation assigned to them. Note that the training data were not annotated in this manner—the classification models used here were not specifically trained for the important publications. Instead, the test data set was annotated in this way in order to evaluate and understand the current systems performance on important studies for the *New Update Alert* task. The number and types of annotations assigned for each topic in the data set are shown in Table 4. Only the 332 included publications out of the 3654 publications in the test set were considered for annotation. After manual review, out of these, only 80 were assigned “important publication” annotations.

**Table 4:** Annotation counts by type and systematic review topic

	**NUMBER OF ANNOTATIONS BY TYPE**					
**TOPIC**	**A**	**P**	**B**	**L**	**M**	**TOTAL**
*ADHD*	1	-	1	-	10	12
*AEDs*	2	-	3	-	-	5
*Antiemetics*	-	-	1	1	15	17
*AtypicalAntipsychotics*	1	8	2	-	-	11
*MSDrugs*	1	-	3	1	-	5
*NasalCorticosteroids*	-	-	-	-	3	3
*OveractiveBladder*	-	1	-	-	-	1
*ProtonPumpInhibitors*	7	1	2	-	16	26
*Sedatives*	-	-	3	-	-	3
***TOTAL***	12	10	12	2	44	**80**

### Classification system

To classify the samples according to whether they should be used to display a *New Update Alert* for each SR, we applied the support vector machine (SVM)-based classification system that we have described in detail in our prior work [10]. Briefly, this is an SVM-based machine learning method that classifies samples based on the signed-margin distance from the separating hyperplane. Samples with large positive margin distances are ranked strongly positive for inclusion, and samples with very negative margin distances are highly ranked as excluded. The cutoff between positive inclusion and negative exclusion predictions is adjustable. Features input to the classifier include uni- and bi-grams, from the title and abstract, and MeSH terms associated with the publication. We use the SVMLight implementation of the SVM algorithm (http://svmlight.joachims.org/), with a linear kernel at default settings [17]. See Additional file 1 online for further details. For this work, publications classified as positive would be used to signal a *New Update Alert*, and those classified as negative would not.

For the *New Update Alert* task, we adjusted the classification cutoff threshold in the following manner. Previously, and in ongoing work, we have studied the user preferences of systematic reviewers in terms of document classification system tradeoffs for New Update Alert. It has been determined that, in general, review experts are more willing to trade off recall for precision for the New Update Alert task, as compared to the work prioritization task that we have previously studied. In particular, the principle investigator of the DERP (one of the senior authors of this paper) consistently preferred a recall of 0.55 and the achievable precision corresponding to that level of recall over all other available levels of recall between 0.99 and 0.55. The context of DERP is important in the choice of 0.55. The team lead would be reviewing multiple topics every month for years – not just a one-off SR every now and then. The level of the continual workload is an important factor. This means that the reviewer found 0.55 as the lowest acceptable recall for this task, leading to the highest precision that the current system can deliver at an acceptable recall. We therefore targeted a recall of 0.55 to study the performance of the classification system on the important publications in each topic.

For each topic, we performed 5 repetitions of two-way cross-validation on the training data, and determined the threshold that lead to a recall of 0.55 for each repetition. These thresholds were averaged together to determine the threshold to use when applying the classifier to the test data set for each topic. Then, for each topic, we trained a classification model on that topic’s training data. We next classified each document in the corresponding test collection, using the computed threshold as a cutoff between a document predicted to raise a *New Update Alert* and a document not predicted to raise an alert. We analyzed the performance of the trained classifiers—both overall, as well as on the designated motivating publications.

## Results

Tables 5, 6 and 7 show the overall performance of the classification models on each of the topics, using the chosen threshold on the training and test sets. While we were able to consistently achieve the target recall of 0.55 on the training sets, recall performance varied widely on the test sets, from a low of 0.134 on *AtypicalAntipsychotics* to a high of 1.0 on *NasalCorticosteroids*. Precision also varied greatly, both on the training data as well as the test set, varying from a low of 0.306 on the *NasalCorticosteroids* test collection to a high of 0.800 on *ProtonPumpInhibitors*.

**Table 5:** Overall, correct, and incorrect alert rates as well as recall of important publications for each topic

**TOPIC**	**PRE-UPDATE PERIOD IN MONTHS**	**OVERALL ALERTS PER MONTH**	**CORRECT ALERTS PER MONTH**	**INCORRECT ALERTS PER MONTH**	**IMPORTANT ARTICLE RECALL**
*ADHD*	15	2.67	1.33	1.33	1.00
*AEDs*	20	1.60	0.65	0.95	0.40
*Antiemetics*	41	0.59	0.24	0.34	0.88
*AtypicalAntipsychotics*	16	1.19	0.56	0.63	0.72
*MSDrugs*	29	0.34	0.21	0.14	0.80
*NasalCorticosteroids*	19	2.58	0.79	1.79	0.33
*OveractiveBladder*	31	1.48	0.68	0.81	1.00
*ProtonPumpInhibitors*	31	0.48	0.39	0.10	0.46
*Sedatives*	19	1.16	0.68	0.47	1.00
***MEAN***	24.56	1.34	0.61	0.73	0.73

**Table 6:** Classifier performance on the training and test sets at the closest threshold to a recall of 0.55 on the training set for each topic

		**TRAINING SET CROSS-VALIDATION**						
**TOPIC**	**THRESHOLD**	**TP**	**TN**	**FP**	**FN**	**Precision**	**Recall**	**F1**
*ADHD*	−0.2466	155	2423	119	130	0.566	0.544	0.555
*AEDs*	−0.3302	57	1361	75	39	0.432	0.594	0.500
*Antiemetics*	−0.3550	70	2196	168	63	0.294	0.526	0.377
*AtypicalAntipsychotics*	−0.1696	375	3544	285	284	0.568	0.569	0.569
*MSDrugs*	−0.2390	77	1699	75	61	0.507	0.558	0.531
*NasalCorticosteroids*	−0.3943	8	61	21	5	0.276	0.615	0.381
*OveractiveBladder*	−0.3461	57	695	128	46	0.308	0.553	0.396
*ProtonPumpInhibitors*	−0.2532	117	2235	98	87	0.544	0.574	0.558
*Sedatives*	−0.2617	79	1583	74	66	0.516	0.545	0.530

True positives (TP), true negatives (TN), false positives (FP), false negatives (FN), F1 measure (F1, the harmonic mean of precision and recall).

**Table 7:** Classifier performance on the training and test sets at the closest threshold to a recall of 0.55 on the training set for each topic

		**TESTING SET**						
**TOPIC**	**THRESHOLD**	**TP**	**TN**	**FP**	**FN**	**Precision**	**Recall**	**F1**
*ADHD*	−0.2466	20	225	20	23	0.500	0.465	0.482
*AEDs*	−0.3302	13	457	19	26	0.406	0.333	0.366
*Antiemetics*	−0.3550	10	320	14	19	0.417	0.345	0.377
*AtypicalAntipsychotics*	−0.1696	9	775	10	58	0.474	0.134	0.209
*MSDrugs*	−0.2390	6	597	4	32	0.600	0.158	0.250
*NasalCorticosteroids*	−0.3943	15	28	34	0	0.306	1.000	0.469
*OveractiveBladder*	−0.3461	21	204	25	4	0.457	0.840	0.592
*ProtonPumpInhibitors*	−0.2532	12	372	3	38	0.800	0.240	0.369
*Sedatives*	−0.2617	13	197	9	13	0.591	0.500	0.542

True positives (TP), true negatives (TN), false positives (FP), false negatives (FN), F1 measure (F1, the harmonic mean of precision and recall).

Figure 1 shows a timeline view of all of the annotated positive samples (studies with a decision to include in the SR update) in the test collection. For each topic, the left end of the timeline shows the end of the prior SR cycle for that topic, and the right end shows the date that the SR update literature search began. The period in between is what we have defined as the *Pre-Update Period*. Each annotation code is represented by a different marker shape, as indicated in the figure legend. Black, filled in markers designate important annotated publications that were correctly recognized by the classification system and therefore could be used to initiate a new update alert (*TP*, true positives). White, unfilled markers represent important, annotated publications that were missed by the classification system and are therefore not able to be used to initiate a new update alert (*FN*, false negatives). Most of the annotated publications are identified by the classification system. Overall there are 57 out of 80 annotated publications identified by the classification system, a recall of 0.7125 on the most important publications. The vast majority of the misses are on articles in the *ProtonPumpInhibitors* topic.

The timeline view shows that there are important differences between the topics. From the figure it is clear that for some topics, substantial new evidence that may motivate a review update begins to accumulate essentially immediately after the prior review is published. This is especially apparent for the topics *ADHD*, *Antiemetics*, and *ProtonPumpInhibitors*. Conversely, some topics do not rapidly accumulate new evidence motivating a review update. *Sedatives* and *NasalCorticosteriods* only have three annotated as important studies during the *Pre-Update Period*, and these topics each only have one publication that would be a year old at the time of the performed review update.

The topics with the most publications annotated as motivating an SR update are *ADHD*, *Antiemetics*, *AtypicalAntipsychotics*, and *ProtonPumpInhibitors*. For *ADHD*, all of the annotated publications are captured for alert. For *Antiemetics*, 15 of 17 motivating publications are correctly predicted. For *AtypicalAntipsychotics*, 8 are correctly predicted and 3 are missed (overlap in the timeline plot obscures some of the points). Finally, for *ProtonPumpInhibitors,* only 12 of the 26 annotated publications are captured for alert. It is interesting to note that for *AEDs* and *ProtonPumpInhibitors* the set of missed publications include not only the M category of generically motivating publications, but the more specific and perhaps more important categories of new or serious adverse events (A) and better designed or novel study (B). For the other topics, the classifier performs well on the more specific annotation categories (A, P, B, and L), and it is only the more general M potentially motivating studies that are missed.

Table 5 summarizes the mean overall, correct, and incorrect alert rates per month, along with recall of important publications, for each of the topics. This can be interpreted under the premise that alerts might be reviewed on a monthly basis. The total number of included (TP) publications (shown in Table 6 and 7) and therefore potentially correct alerts, as well as the number of motivating publications (shown in Table 4) varies widely across topics. However, from a practical point of view, the actual correct and incorrect alert rates shown in Table 5 do not vary much. The alert rates range from a low of about 0.50 alerts per month for *ProtonPumpInhibitors* to a high of 2.67 alerts per month for ADHD. The number of correct alerts exceeds the number of incorrect alerts on three topics, has about the same number of correct and incorrect alerts on one topic, and a higher number of incorrect alerts on six topics. However, the imbalance between correct and incorrect alerts is understandable, since included documents are a small percentage of documents returned by the original query. The relative frequency of excluded documents is typically vastly higher than included documents for SR topics (see Table 2). Because of this, for the New Update Alert task, the alert rate for the included and important publications, combined with the absolute number of incorrect alerts is a more relevant metric of performance than a comparison between the correct and incorrect alert rate.

While the number of update-motivating publications annotated for each topic varies quite a bit, the overall rate of alerts that need to be monitored is small, with most of the motivating publications recognized and leading to a correct alert. For example, *OveractiveBladder* only has one annotated publication during the pre-update period, and this publication is correctly recognized by the classifier. The annotation type of this publication is P, a new patient population subgroup, indication, or comorbidity. From Table 7 it can be seen that, out of 254 potential publications in the test set for this topic and a test set precision of 0.457, 46 alerts would be initiated over the 31 month time period. Twenty-one of these alerts would be true positives and 25 of the alerts would be false positives. One of the true positive alerts would be for the update motivating publication. Over the 31 month pre-update period, this works out to about 1.5 alerts per month, with a true alert occurring approximately every 1.5 months, and a false alert occurring about every 1.25 months.

## Discussion

While there are noticeable differences between the topics, in terms of the performance of the classifier, these differences do not seem to translate into large practical differences in the overall rate of the *New Update Alerts*, nor in the overall rate of correct alerts or false alarms. The per-month alert rates are low, which implies that the overhead of monitoring these alerts would also be low. While the precision performance of the classifier is far from perfect, the large numbers of negative publications captured by the SR topic query means that moderate precision performance results in filtering out quite a large number of these false negatives – preventing them from signalling an alert.

The recall of the classification system is also far from perfect. However, the vast majority of the update motivating publications – the most important to recognize as *New Update Alerts* – are identified by the classifier, and are therefore available to initiate an alert. The overall recall of the important publications is 0.73. This is good enough to recognize one or more update motivating publications for each topic at least 6 months before the beginning of the scheduled review update. The number of new articles in an SR topic is not nearly as important for scheduling an update as the impact of the information in specific articles. A single important article may be enough to motivate a review update. This will occur if the evidence in the article changes the recommendations or strength of conclusions in the SR. Conversely, the publication of many new articles in a topic that do nothing but reiterate previously existing evidence may not motivate a review update, as the recommendations or strength of conclusions in the SR are much less likely to change based on those articles. Therefore, while the performance of the system certainly would benefit from additional research and development, the ability to focus reviewer attention on publications within an SR topic that individually motivate a review update is a useful property of the current system.

Even given the variations in performance across topics, a notable number of annotated publications are captured for alert for all topics. This is accomplished with an overall low alert rate. We think that, given the rate of alerts we found, an SR expert using a live alert system could quickly review the alerts, identify from this set the important publications, and use this information in planning and scheduling review updates. This information could also be shared with agencies funding SRs and updates, to provide context and motivation at the appropriate time when a topic has new evidence that needs to be incorporated into the SR in order to better and more efficiently support the practice of EBM.

Furthermore, this kind of information could be useful for prioritization of review updates between different topics. It may be necessary to make tradeoffs considering which of a number of SR topics are most in need of update. Expert SR resources are limited, and it seems reasonable to update the topics that have not only a large number of relevant publications, but, more significantly, a number of important publications. The most important publications add new information to the evidence base for a given topic. These publications are the ones that are most likely to inform the medical community about new indications or potential harms, and influence the conclusions or recommendations of an evidence report or meta-analysis. New update alert information could be used to prioritize one review update over another, based on the newly published information in each of these areas, and the level of importance of this information to the medical community. At the current level of performance we expect that our approach will be most useful to the senior reviewer or leader of an SR team. The senior review team lead will be in the best position to effectively combine their domain expertise and other SR topic knowledge with results of our system to best determine when to schedule a review update.

For example, looking back at the timeline for *OveractiveBladder*, there is only one publication designated as important according to the annotation schema. This publication is marked with category M, “potentially motivating for review update”, the most general, and typically the weakest of the annotation categories. On the other hand, for *ProtonPumpInhibitors* new relevant publications start becoming available almost as soon as the original report is published. The report for this topic is at risk of quickly becoming out of date, especially because the new publications represent several of the annotation categories. If a choice about assigning SR update resources needed to be made, it would be reasonable to assume that *ProtonPumpInhibitors* would have a higher priority than *OveractiveBladder*. Note that this is true for this example even given that both topics have a pre-update period of about two and a half years - about half the median lifespan of an SR found by Shojania and colleagues as described in the Introduction. The update for *ProtonPumpInhibitors* was likely needed immediately, while the *OveractiveBladder* update perhaps could have been postponed.

This study has several limitations and opportunities for future work. First and foremost, as far as generalizability is concerned, the work was done using the publication inclusion decisions from a single SR group, and the only topics that were available to be studied were those with a review completed by the DERP as well as a completed update within the time window of our study. All of these SRs performed by the DERP focus on drug therapy, and certainly there are a wide range of other topics for which there are, and need to be, SRs. Future work should include studying a larger set of SR teams, as well as a more diverse set of SR topics.

Secondly, the classification system was not specifically optimized in any way for the most important publications for new update alert. In this article we are proposing a new classification task, stating why it is important, and how it can be used to improve the EBM process. While the present system does identify most of the publications annotated as important for each topic, it did tend to miss articles with certain annotations more than others. There are significant numbers of misses, particularly in the *AEDs* and *ProtonPumpInhibitor* topics.

Therefore, with this initial work, we hope to motivate future research on this task. It should be possible to train a classification system to recognize specific features corresponding to the motivating publication annotation types, and to more highly rank publications with these features. For example, within the group of important publications that were missed by the classifier, studies including new adverse events were often missed. This represents a promising avenue for future work and optimization. The annotation scheme that we have developed here could be used to create a training set optimized to recognize these specific categories of important publications. Adverse events could be specifically recognized and their presence incorporated into a model that scores publications more positively for including these adverse events. Furthermore, since novel evidence is an important part of why a particular article may motivate an SR update, it may be useful to specifically recognize new forms of evidence. For example, this would include data such as previously unreported or unstudied adverse events within the literature for an SR topic.

Finally, the recall and precision of the classifier varied much more widely in the test set than in the cross-validation estimates obtained on the training set. We attribute at least some of this variation to the small sample sizes in the test sets. For the largest test set topic, *Sedatives*, the achieved recall of 0.50 is reasonably close to the target recall of 0.55, and the achieved precision of 0.591 on the test set is reasonably close to that predicted on the training set, 0.516. Further study will be required to determine whether additional effects, such as topic drift [18,19] (the change in the language or essential concepts within topic discourse over time), are also coming into play here.

## Conclusions

This work is an initial analysis of the opportunities and challenges in aiding the SR update planning process with an informatics-based machine learning approach. We have demonstrated that automated document classification has the potential to be useful in the period between the publication of an SR and the beginning of the literature search for the next update for that review, a period we termed the *Pre-Update Period*. We have defined a classification task useful to the SR processing during this time period, called *New Update Alert*, and studied the performance of current machine learning models to the significant articles published during this time period for nine SR topics. In terms of their potential to motivate a review update, some publications are more important than others, and contribute specific types of new knowledge to the topic evidence base. Therefore we have designed and applied an annotation schema to identify and characterize the publications particularly important in motivating the need for an SR review. Finally, we have analyzed the performance of our pre-existing classification system on these review-update motivating publications, and identified important areas for future improvement and optimization.

The system proposed here could be used continually after a topic is completed, with very little additional manpower required. This would provide a clear indication on which topics need updating before the typical two-year cycle, and which are unlikely to need it. This 1) saves time on the part of reviewers, 2) reduces time delays in updating topics that develop faster, and 3) prevents time and effort spent on reviewing topics not yet in need of an update. The system fits in well with the current RAND and Ottawa approaches, serving as a continuous prior step before the decision is made to allocate the substantial resources required by these approaches. *New Update Alert* has the potential to change how SR review resources are scheduled, planned, and allocated, and future work will further study how to best incorporate this approach into the overall SR planning workflow.

## Competing interests

The authors declare that they have no competing interests.

## Authors’ contributions

All authors contributed to the construction of the SR datasets. AMC and MM designed and applied the annotation guide. AMC and KA built the automated text processing system and ran the machine learning experiments. All authors contributed to the writing and to the editing of the manuscript. All authors read and approved the final manuscript.

## Pre-publication history

The pre-publication history for this paper can be accessed here:

http://www.biomedcentral.com/1472-6947/12/33/prepub

## Supplementary Material

Additional file 1 **Appendix 1 for Studying the Potential Impact of Automated Document Classification on the Systematic Review Update Scheduling Process**. Click here for file
