# Supplementary material for: Studying the potential impact of automated document classification on scheduling a systematic review update
Source: BMC Med Inform Decis Mak. 2012 Apr 19;12:33. doi: 10.1186/1472-6947-12-33 (PMC3420236; doi:10.1186/1472-6947-12-33)
Supplement: Additional file 1 — Appendix 1 for Studying the Potential Impact of Automated Document Classification on the Systematic Review Update Scheduling Process. [file 1472-6947-12-33-S1.docx]

**Appendix 1 for Studying the Potential Impact of Automated Document Classification on the Systematic Review Update Scheduling Process**

Detailed description of the text classification algorithm

For the classification task described in the main manuscript, we applied a support vector machine (SVM) based classifier, using features derived from the title, abstract, and MEDLINE MeSH terms associated with each article. We used the SVMLight (http://svmlight.joachims.org/) implementation of SVM with the feature sets as described below. We used the default settings for SVMLight with a linear kernel, including using the default value of the C tuning parameter. This parameter allows for adjustment of the trade off between the width of the separating margin, and classification errors on the training data. We have done extensive past work on systematic review text classification with SVMLight, using grid search and cross-validation to investigate the impact of adjusting tuning parameters. We have found that there is little impact in adjusting the C tuning parameter with text classification problems involving many more features than training samples, such as is the case here. With the large numbers of features typically used in text classification, training error is minimal, and the size of the C parameter has little impact. The default value of one over the average of the sample norm usually works well. Un-validated grid search on the training data may result in misleading, over-trained results. Additionally, using the default value of C reduces the need for tuning and therefore eliminates the need for additional tuning data or reusing the training data for tuning, which may bias other aspects of an evaluation, such as feature selection.

The features used in our classifier include those based on title and abstract tokens and bigrams, as well as MeSH terms. The title and abstract text were combined, converted to lowercase, and then processed using a simple tokenizer that separates tokens based on whitespace and punctuation. Uni-gram and bi-gram token features were then extracted from the token stream, and bi-gram features were normalized by sorting its component uni-grams in alphabetical order. For features based on MeSH terms (MESH), we included features representing the presence or absence of primary terms, main terms and subheadings separately, as well as complete MeSH terms as given in the MEDLINE record.

All features of the above types were input to the SVM classifier. Neither stemming nor feature selection was performed. All features were encoded as binary terms in the feature vector; a value of 1.0 was assigned if the feature was present, and a value of 0.0 was assigned for absent features.

We addressed the class skew problem (many negative excluded samples, few positive included samples) by first ranking the test samples according to the signed margin distance produced by applying the SVM linear weight vector to the sample vectors using the dot product. The signed margin distance can vary from large positive numbers, indicating a strong positive prediction of included, to a very negative number, indicating a strong prediction of excluded. To create the final prediction for each sample we applied a cut-off value on the signed margin distance. Values above the cut-off were predicted to be positive, values at or below were predicted to be negative. Applying cross-validation to the training data, we identified the signed margin threshold value that resulted in a recall of 0.55. This value of recall was found to be the favored recall/precision tradeoff in prior work studying reviewer preferences for the new update alert task.
